# Mapping the antileishmanial structure-activity relationship of eugenol

**DOI:** 10.3389/fphar.2026.1923146

**Published:** 2026-09-30

**Authors:** Lucas Daut, Jonathan Taylor, Nicola S. Carter, Brendan D. Stamper

**Affiliations:** Pharmaceutical Sciences Department, Pacific University School of Pharmacy, Hillsboro, OR, United States

**Keywords:** antileishmanial activity, chavicol, eugenol, Leishmania donovani, Leishmaniasis, natural products, pharmacology

## Abstract

Leishmaniasis is a neglected tropical disease with a devastating global impact. The current medications on the market for treating this protozoan parasitic infection are insufficient to fit the global need, with long durations of therapy, growing resistance rates, and severe adverse effects. This investigation aims to characterize an antileishmanial structure-activity relationship for the natural product eugenol. With *Leishmania donovani* as a model, a series of dose-response curves were performed against eugenol and several of its structural analogs. The same set of compounds was tested against the human monocyte cell THP-1 to characterize their selective toxicity. Subsequently, the pharmacokinetic parameters of the tested compounds were estimated using the *in silico* modelling software, Percepta®. Eugenol and its analogs demonstrated varying levels of antileishmanial potency, with 4-allylphenol (chavicol) as the most potent compound tested and with the most promising selectivity index. In comparing the potencies and structures of the compounds tested, the data suggests that the para-substituted allyl tail and the phenolic group are important to conferring potency, while the ortho-methoxy substitutions decreased potency. Furthermore, the pharmacokinetic properties of these compounds were predicted to closely resemble eugenol with little risk of cytochrome P450 (CYP) interactions. While more testing is needed to confirm the mechanisms and safety of these compounds, these results suggest eugenol analogs show promise as future antileishmanial therapies.

## 1 Introduction

Leishmaniasis is a neglected tropical disease caused by the vector-borne parasites of the genus *Leishmania*. Though its impact in the US is small, the World Health Organization (WHO) reports over 12, 000, 000 individuals infected with leishmaniasis worldwide and 1 billion individuals at risk in endemic areas (Ansari et al., 2025). Annually, the WHO estimates 0.7 to 1 million new cases, and 20,000 to 30,000 deaths, mostly arising from visceralizing infections (Alvar et al., 2012). This disease affects nearly 100 countries worldwide, with the highest prevalence found in tropical countries such as Brazil, India, and Somalia. *Leishmania*, like many neglected tropical diseases, has multiple biological reservoirs and vector species, rendering it challenging to control (Pareyn et al., 2025; Burza et al., 2018).

The disease presents with different types of symptoms based on the infecting species, which can be generally categorized as visceral, cutaneous, or mucocutaneous leishmaniasis. While the majority of new cases are cutaneous, visceral leishmaniasis (VL) is responsible for the majority of disease-related deaths (Aronson et al., 2016; Carter et al., 2021).

The only confirmed vector for transmitting leishmaniasis is the sandfly. Unlike mosquitos, sandflies are much smaller, creating additional challenges in vector management. There is currently no human vaccine available for leishmaniasis and it remains a persistent global health challenge despite WHO-led efforts.

There are several available medications to treat VL, however, only miltefosine and liposomal amphotericin B are currently approved by the U.S. Food and Drug Administration (FDA). Other medications used to treat leishmaniasis include paromomycin, sodium stibogluconate, amphotericin B (deoxycholate or lipid complex), and pentamidine. These medications carry serious side effect profiles, require long durations of therapy, and several of them are very expensive, adding barriers to patient access and adherence (Pareyn et al., 2025). Another challenge to using these agents is the continuously growing resistance rates to these therapies (Carrasco-Martin et al., 2025; Enbiale and Dassoni, 2025; Omwenga et al., 2026). Even without developed resistance, these medications do not reliably lead to a sterile cure. The need for such long durations of therapy, the adverse effect profile, and the cost of these medications create this barrier to patient adherence (Sundar et al., 2000).

Based on the cost, toxicities, barriers to access, and growing resistance, the need for novel antileishmanial agents in the modern world is clear. Natural medicines have been used to treat infections like leishmaniasis for hundreds of years. A notable example of the efficacy of natural medicine to treat parasitic infections is the use of quinine for malaria. Quinine, an extract from cinchona bark, was one of the first antimalarials to be broadly used (Tagboto and Townson, 2001). Subsequently, many structural derivatives of quinine, including chloroquine and mefloquine, among others, were developed to increase the efficacy and pharmacokinetic parameters of quinine (Spikes, 1998). In the current era, artesunate, a derivative of the natural product artemisinin, is the standard therapy for treating malaria. Artemisinin is a natural extract derived from sweet wormwood (Kingston and Cassera, 2022; Swamikannu et al., 2024), and also has been shown to have activity against different *Leishmania* sp. (Sarkar et al., 2024).

Several studies have been conducted on the antileishmanial activity of natural products, like artemisinin (Carter et al., 2021; Kowalczyk et al., 2020; Le et al., 2017; Oryan and Akbari, 2016; Sarkar et al., 2024). In one study, investigators explored the antileishmanial properties of native Vietnamese plant essential oils and then characterized the chemical components of the most potent candidates. Interestingly, in this investigation, one of the top-performing essential oils was Ocimum gratissimum (African basil), which is comprised of over 86% eugenol (Le et al., 2017). Other studies investigated the activity of thymol, the primary component of thyme essential oil, and a simple phenol that structurally resembles eugenol. Investigations into the mechanism of action of natural phenolic compounds like eugenol and thymol have yielded a variety of potential mechanisms including reactive oxygen species generation, apoptotic signal ing, and immune recruitment (Charan Raja et al., 2021; da Silva et al., 2018; de Morais et al., 2014; Hughes et al., 2023; Sova and Saso, 2020). Since resistance to standard antileishmanial treatments is ever-growing, gaining a better understanding of how natural compounds exert their antileishmanial effects bodes well for their potential as future therapies for this disease.

While the bioactive potential of alkaloids and glycosides is well-recognized (Sasidharan et al., 2011), natural polyphenols offer a particularly promising avenue for targeted antileishmanial therapy (Carter et al., 2021). In the present work, we compared the activity of eugenol and several commercially available eugenol analogs against both major life stages of *L. donovani* and evaluated cytotoxicity in THP-1 cells to establish a structure-activity relationship (SAR) and reveal molecular regions most amenable to modification for future drug development. An additional *in silico* screening of the pharmacokinetic parameters of eugenol and its analogs was also conducted to evaluate their potential for metabolism and *in vivo* behavior, with the overarching goal to evaluate the potential of a eugenol-based compound as a future antileishmanial agent.

## 2 Materials and methods

### 2.1 Key chemical and reagents

For a complete list of reagents used please see Supplementary Table S1.

### 2.2 Cell culture

Wild-type strain 1S2D LdBob *L. donovani* promastigotes (Dwyer, 1976; Goyard et al., 2003), provided by Dr. Stephen M. Beverley (Washington University, St. Louis, MO), were cultured at 27 °C with 5% CO_2_ in Dulbeccos Modified Eagles medium modified for *Leishmania* cultivation supplemented with 10% heat-inactivated fetal bovine serum (Goyard et al., 2003; Martin et al., 2014). For the experiments with axenic amastigotes, late-stage stationary phase cultures of promastigotes were transformed by placing 0.05 mL of culture into 4.5 mL of amastigote media (Goyard et al., 2003). Cultures were incubated at 27 °C with 5% CO_2_ for 48–72 h, before transitioning to 37 °C. Transformed axenic amastigote cultures were maintained at 37 °C with 5% CO_2_ in amastigote media, with the media refreshed every 3–4 days (Goyard et al., 2003).

THP-1 human monocyte cells, a suspension human leukemia monocyte cell line (Tsuchiya et al., 1980), were obtained from the American Type Culture Collection (ATCC, TIB-202) and were cultivated as recommended in RPMI 1640 media supplemented with 10% heat-inactivated FBS (Neuromics, FBS002) at 37 °C. Cells were maintained in this medium at a density of between 1 × 10^5^ to 1.5 × 10^6^ cells/mL.

### 2.3 Compound screening and dose-response analyses

Stock solutions of all eugenol SAR compounds were prepared at 100 mM in ethanol, which was selected for its favorable solubility profile and matrix miscibility. Working solutions were prepared in destination media at 2x the highest target concentration, maintaining a maximum final ethanol concentration of 0.2% (v/v) to avoid cellular toxicity and assay interference. Two-fold serial dilutions were performed across 96-well plates. Assays were initiated by adding 100 µL of cell suspension to 100 µL of drug solution per well (final volume per well of 200 µL).

For *Leishmania* promastigotes and axenic amastigotes, cells were diluted to a final density of 1 × 10^6^ cells/mL (2 × 10^6^ cells/mL initial concentration) and incubated at 27 °C (promastigotes) or 37 °C (amastigotes) with 5% CO_2_. For THP-1 cells, cell suspensions were prepared in RPMI-1640 media supplemented with 10% heat-inactivated fetal bovine serum and seeded at a final density of 1 × 10^5^ cells/mL (2 × 10^5^ cells/mL initial concentration) and incubated at 37 °C with 5% CO_2_.

Following a 48 h incubation, 10 µL of 20x resazurin stock (0.88 mM in PBS) was added per well and incubated with cells for 24 h. Resazurin is a non-toxic metabolic dye whose reduction to the fluorescent product resorufin is proportional to the number of viable cells in the well (Mikus and Steverding, 2000). The proportion of resazurin converted to resorufin was quantified by fluorescence λ_ex_ = 554 nm, λ_em_ = 593 nm on a BioTek Synergy H1 Multimode Reader (Agilent, Santa Clara, CA). All compounds were evaluated in ≥3 biological replicates, each containing 6 to 8 technical replicates per run.

### 2.4 Data visualization and statistical analyses

Dose-response curves were generated by log-transforming drug concentrations and normalizing to the untreated control (100%). Data were fitted to a non-linear regression model (log [inhibitor] vs. normalized response, variable slope) using GraphPad Prism (version 11.0.0; GraphPad Software, San Diego, CA, USA) to determine IC50 values. To statistically analyze the resulting IC50 and LC50 data a two-sample t-test assuming unequal variances was used to directly compare datasets for significance.

### 2.5 Pharmacokinetic investigation

To evaluate the pharmacokinetics of eugenol and its analogs, a screen of all the compounds using Percepta® (ACD/Labs; 2019.1.1 Build 3200. 17 July 2019) was conducted. Percepta® is an experimentally trained software program capable of predicting small molecule/protein interactions that has demonstrated utility across various *in silico* applications (Kalliokoski and Sinervo, 2019; Spaenig et al., 2026). This software estimates cytochrome P-450 (CYP) substrates, CYP inhibitors, P-glycoprotein (P-gp) substrates, LogP (partitioning coefficient), protein binding and more. Using this tool, all tested compounds were evaluated for potential CYP interactions, P-gp interactions and other common pharmacokinetic parameters that have implications in the feasibility of these compounds to be developed as pharmaceuticals.

## 3 Results

### 3.1 Eugenol SAR screen against *L. donovani* promastigotes and amastigotes

A structure-activity relationship for eugenol was undertaken to identify critical moieties that promote antileishmanial activity as well as regions that might benefit from structural modifications to guide future drug design efforts. In screening eugenol against the *L. donovani* promastigotes, we calculated an IC50 of 0.639 mM ± 0.083 (Table 1, Supplementary Figure S1). Several of the eugenol structural derivatives had lower calculated IC50 averages than eugenol including 4-allyl-2,6-dimethoxyphenol, 2,6-dimethoxyphenol, 4-allylphenol, 3-allyl-4-hydroxyacetophenone, and 2-allyl-6-methoxyphenol (Table 1).

**TABLE 1:** Dose-response curves for various eugenol analogs against *L. donovani* promastigotes and axenic amastigotes.

Compound	Average IC50 ± SD (mM)	
	Promastigote	Amastigote
Eugenol	0.639 ± 0.083 (n = 7)	0.108 ± 0.024 (n = 4)
4-Allyl-2,6-dimethoxyphenol	0.536 ± 0.110 (n = 3)	0.138 ± 0.049 (n = 3)
2-Methoxyphenol	1.049 ± 0.069 (n = 3)*	0.089 ± 0.008 (n = 3)*
2,6-Dimethoxyphenol	0.447 ± 0.105 (n = 3)	0.328 ± 0.085 (n = 3)
4-Allylphenol	0.222 ± 0.026 (n = 3)*	0.048 ± 0.025 (n = 3)*
4-Allyl-1,2-dimethoxybenzene	0.877 ± 0.231 (n = 3)	0.157 ± 0.044 (n = 3)
3-Allyl-4-hydroxyacetophenone	0.585 ± 0.346 (n = 3)	0.099 ± 0.065 (n = 3)
2-Allyl-6-methoxyphenol	0.292 ± 0.190 (n = 3)*	0.085 ± 0.031 (n = 3)

* *p*-value <0.05 for IC50 when compared to eugenol IC50 against promastigote or amastigote lifestages.

Of these compounds, 4-allylphenol demonstrated the highest potency against promastigotes in culture with an average IC50 of 0.222 ± 0.026 mM (Table 1, Supplementary Figure S1). This difference in IC50 between 4-allylphenol and eugenol was statistically significant ( P <0.05) as was the difference between eugenol and the next highest potency compound (2-allyl-6-methoxyphenol, P <0.05). 2-Methoxyphenol and 4-allyl-1,2-dimethoxybenzene demonstrated weaker antileishmanial potency compared to eugenol.

In the set of dose-response curve experiments conducted in axenically cultured amastigotes, all compounds demonstrated higher potency compared to their respective IC50 results against promastigote parasites (Table 1, Supplementary Figure S2). In this set of experiments, once again, 4-allylphenol had the greatest antileishmanial potency, with an average amastigote IC50 of 0.048 ± 0.025 mM (Table 1).

### 3.2 Eugenol SAR toxicity screen against THP-1 monocytes

LC50 values were also determined against THP-1 human monocytes to compare relative toxicities between a human monocyte host cell line and the wild-type *L. donovani* cells. In the set of dose-response curves prepared with the THP-1 monocytes; 2,6-dimethoxyphenol demonstrated the highest potency with an average LC50 of 0.505 ± 0.220 mM but this was not statistically different from the LC50 of eugenol 0.568 ± 0.06 mM ( P = 0.376) (Table 2, Supplementary Figure S3). Notably, 2,6-dimethoxyphenol showed the least potency against axenic amastigotes. In fact, the majority of compounds showed similar LC50 values of between 0.505 and 0.619 mM. The two exceptions being 4-allyl-1,2-dimethoxybenzene (LC50 of 1.256 ± 0.311 mM), and 2-methoxyphenol, for which an LC50 value could not be assessed due to the limits of solubility.

**TABLE 2:** LC50 for eugenol analogs against THP-1 monocytes.

Compound	Average LC50 ± SD (mM)
Eugenol	0.568 ± 0.061 (n = 3)
4-Allyl-2,6-dimethoxyphenol	0.542 ± 0.032 (n = 3)
2-Methoxyphenol	ND
2,6-Dimethoxyphenol	0.505 ± 0.220 (n = 3)
4-Allylphenol	0.578 ± 0.014 (n = 3)
4-Allyl-1,2-dimethoxybenzene	1.256 ± 0.311 (n = 3)
3-Allyl-4-hydroxyacetophenone	0.548 ± 0.010 (n = 3)
2-Allyl-6-methoxyphenol	0.619 ± 0.014 (n = 3)

ND, not determined due to solubility limitations (>2 mM).

The selectivity indices for the screened eugenol analogs were calculated by dividing the average LC50 values calculated for THP-1 monocytes by the average calculated IC50 values for *L. donovani* promastigotes or axenic amastigotes. The generic criteria for determining whether a compound is selective against an infectious disease is based on parameters established by the Global Health Innovative Technology (GHIT) Fund (Katsuno et al., 2015). Typically, compounds must demonstrate at least a >10-fold selectivity index relative to mammalian cell cytotoxicity to be considered for further evaluation and lead development. Promastigote selectivity indices ranged from 0.889 (eugenol) to 2.608 (4-allylphenol). While axenic amastigote selectivity indices ranged from 1.539 (2,6-dimethoxyphenol) to 12.049 (4-allylphenol) (Table 3). Again, for 2-methoxyphenol, the selectivity indices could not be determined due to limits of solubility for this compound in the assay precluding the determination of an LC50 value.

**TABLE 3:** Comparison of the IC50 and LC50 values for various eugenol analogs against *L. donovani* and THP-1 cells and calculation of a selectivity index.

Compound	Average IC50 (mM)		Average LC50 (mM)	Calculated selectivity index	
	Promastigote	Amastigote	THP-1 cells	Promastigote	Amastigote
Eugenol	0.639	0.108	0.568	0.889	5.270
4-Allyl-2,6-dimethoxyphenol	0.536	0.138	0.542	1.011	3.941
2-Methoxyphenol	1.049	0.089	ND	–	–
2,6-Dimethoxyphenol	0.447	0.328	0.505	1.130	1.539
4-Allylphenol	0.222	0.048	0.578	2.608	12.049
4-Allyl-1,2-dimethoxybenzene	0.877	0.157	1.256	1.432	7.981
3-Allyl-4-hydroxyacetophenone	0.585	0.099	0.548	0.937	5.530
2-Allyl-6-methoxyphenol	0.292	0.085	0.619	2.121	7.256

ND, Not Determined due to solubility limitations (>2 mM).

In comparing promastigote and amastigote potency, with that against THP-1 cells, 4-allylphenol demonstrated the strongest leishmanial selectivity index with calculated values of 2.608 and 12.049, respectively (Table 3). Notably, with the exception of 4-allylphenol and 2-allyl-6-methoxyphenol, none of the compounds had a calculated selectivity index above 2 when comparing potency against promastigotes with THP-1 cells. In contrast, with the exception of 2,6-dimethoxyphenol, all of the compounds had calculated selectivity indices ≥3.9 against axenic amastigotes.

### 3.3 Pharmacokinetic predictions for eugenol and its analogs

Percepta® software was utilized to make a number of predictions about the pharmacokinetic properties of eugenol and its derivatives. These properties included cytochrome P450 (CYP450) interactions, LogP, and protein binding (%), and were specifically selected due to their possible effect on elimination half-life or drug-drug interaction potential. No experimental data from our lab was programmed into the Percepta® in order to generate an unbiased output from the program (Supplementary Table S2). As expected based on their structural similarities, Percepta® predicted eugenol to have similar pharmacokinetic properties to its structural analogs overall with logP values in the mid-2s, protein binding % in the mid-70s and low or no probability/reliability to serve as P450 substrates and inhibitors. The only exceptions to this generalized profile were 2-methoxyphenol and 2,6-dimethoxyphenol, which were predicted to have lower LogP values compared to the others (1.34 and 1.12, respectively).

## 4 Discussion

Eugenol is a phenolic compound found in many aromatic spices and already has documented human use, namely, as an antiseptic and numbing agent in dental procedures. There is also literature supporting the safety and benefits of eugenol (Pramod et al., 2010; Taleuzzaman et al., 2021). A growing body of evidence supports the antileishmanial activity of eugenol, with one study reporting an IC50 of 4.15 mM (Sharma et al., 2017). Possible mechanisms of eugenol’s antileishmanial activity have been investigated both *in vitro* and *in vivo*, and point to ROS-mediated apoptosis, cell membrane disruption, lipid storage, and immune modulation (Hughes et al., 2023; Kar et al., 2021; Misra et al., 2009). However, there is a gap in understanding regarding what structural components of eugenol contribute to its mechanism. The information provided here surrounding eugenol’s antileishmanial pharmacophore will help to better inform both lead development and a deeper mechanistic understanding.

A number of observations can be made following the eugenol antileishmanial SAR that was conducted in promastigotes. First, based on the slight decrease in potency demonstrated by 4-allyl-1,2-dimethoxybenzene compared to the other compounds, we can infer that the presence of an exposed phenol group confers antileishmanial potency. This is consistent with findings in the literature where compounds such as quercetin, catechin, and gallic acid have shown promise as antileishmanials (Carter et al., 2021). However, the difference in potency between 4-allyl-1,2-dimethoxybenzene and eugenol in promastigotes and axenic amastigotes was not statistically significant (P = 0.607) (Table 1). The noted activity of an exposed phenol may also hint at one mechanism of killing. Phenol groups have been shown to possess prooxidant activity under the right conditions, leading to oxidative stress and cell death (Nowak et al., 2022). Species within the *Leishmania* genus including our model organism *L. donovani* are particularly susceptible to oxidative stress due to the structure of their single mitochondrion (De Sarkar et al., 2019).

Though it is not consistent throughout the promastigote dataset, the addition of the allyl chain seems to augment antileishmanial potency. This increase in potency is well highlighted when comparing 4-allylphenol to the other compounds (Table 1). In several cases, this difference was not statistically significant due to variation in the data. The increased potency could be explained by the ability of the allyl chain to contribute electron density through induction effects even though the olefin is not in resonance with the aromatic ring.

When we begin looking at the amastigote data, we see similar trends to those observed in the promastigotes. First, 4-allyl-1,2-dimethoxybenzene appears to have reduced potency when compared to eugenol. The removal of the allyl tail generally appears to decrease the potency of these compounds with 2,6-dimethoxyphenol being significantly less potent than eugenol. The importance of the allyl tail is further verified within the amastigote dataset by the potency of 4-allylphenol compared against the two compounds lacking this tail (2-methoxyphenol and 2,6-dimethoxyphenol). Our results are consistent with those from a similar SAR study that evaluated dehydrodieugenol B analogs against *Leishmania infantum* (Amaral et al., 2023). While not directly comparable due to differences in methods, *Leishmania* species, and chemical structure, this research group found that saturation or deletion of an allyl sidechain in dehydrodieugenol B, analogous to eugenol’s, did not generally lead to antileishmanial activity. One exception was substitution of the allyl group with specific polar moieties, which did increase antileishmanial activity, and would be worth exploring in future studies with eugenol.

When comparing leishmanial potency to THP-1 toxicity, we see that most compounds are more toxic to THP-1 cells than they are to promastigotes. We can also see that most compounds are more potent in amastigotes than either the THP-1 cell line or the promastigotes. If we continue with the assumption that these compounds are pro-oxidants in *L. donovani* and exhibit leishmanial toxicity through oxidative stress, it is reasonable to suggest that amastigotes are more susceptible to this type of stress than promastigotes because most of the tested compounds were more toxic to these cells. Though it is not certain why, the literature does support both intracellular and axenic amastigotes being more sensitive to oxidative stress based on ROS-generating antileishmanials (Brígido et al., 2025). It is also plausible that these compounds are not causing oxidative stress in the THP-1 cell line, explained by the reduced variation in LC50s between compounds.

When comparing the structure of 4-allylphenol to the other tested compounds, the primary structural difference is the absence of any ortho substitutions to the phenol group on the ring. This lack of ortho substitutions coupled with its relative potency could suggest some steric hindrance of the interacting phenol. However, if steric hindrance is affecting antileishmanial potency we might expect eugenol to be more potent than 4-allyl-2,6-dimethoxyphenol, which was not observed.

To parse out the role of sterics the eugenol structure-activity relationship testing other eugenol analogs, like 2-allylphenol or 2-allyl-4-methoxyphenol, could provide clearer insight. Additionally, testing phenol alone would provide interesting insight into the impact of the allyl tail. It is worth noting here that phenol has documented toxicity to mammalian cells and would not be viable as an antileishmanial agent; thus, this data would be purely to quantitate the effect of an allyl ring substitution (Phenol, 1999).

In future investigations, using 4-allylphenol as the primary compound could yield more promising results based on what was observed. For example, creating a structural conjugate to this compound could further improve antileishmanial activity. 4-allylphenol is a naturally occurring volatile compound, also known as chavicol that is commonly used as an odorant and flavoring agent (Api et al., 2022). An investigation into the toxicity of 4-allylphenol demonstrated some risk for systemic toxicity at high doses, but no risk of genotoxicity and minimal risk for respiratory effects, photoirritation, or skin sensitization (Api et al., 2022). In one study, 4-allylphenol was noted to have free radical scavenging activity with an EC50 of 7.5 mM using a DPPH assay (Murakami et al., 2015). Structural differences between human and leishmanial cells may be paramount in the selective toxicity of 4-allylphenol, with the compound acting as a radical scavenger in mammalian cells, but a pro-oxidant in parasites. Natural products often have numerous biological targets making it difficult to characterize the full scope of their activity. However, based on the results of the SAR we believe that one potential antileishmanial mechanism that eugenol and its derivatives exhibit are pro-oxidant properties in *L. donovani* (Misra et al., 2009). Eugenol and many other natural compounds that are widely considered to be antioxidants may have a paradoxical pro-oxidant effect under the right circumstances.

One investigation suggested that the antileishmanial mechanism of eugenol was apoptosis in response to oxidative stress (Misra et al., 2009). There are several potential ROS assays that could be employed to test eugenol’s ROS generation potential in *Leishmania*. One of the more common ROS assays uses the probe dichlorofluorescein diacetate (DCFDA). DCFDA is taken up by metabolically active cells where it is metabolized to the metabolite 2',7'-dichlorodihydrofluorescein. Once this compound comes into contact with ROS, it then reacts and is converted into the fluorescent compound dichlorofluorescein (DCF) (Eruslanov and Kusmartsev, 2010).

If it is demonstrated that the antileishmanial mechanism of action of eugenol and its analogs is mediated through oxidative stress to the parasites, testing these compounds with a second known ROS-generating agent could improve parasite killing. Additionally, preparing a set of experiments that combine eugenol with the classic antileishmanials may produce synergistic killing; for example, antimonial drugs, which act by disrupting trypanothione metabolism, the major antioxidant defense system in these parasites (Wyllie et al., 2004). Having multiple mechanisms working against this infection may also improve cure rates. In one study, investigators dosed eugenol oleate with liposomal amphotericin B, which resulted in a synergistic effect against *L. donovani* promastigotes. In this investigation they also noted improved nitrate generation and increased proinflammatory cytokines in infected macrophages with the eugenol oleate and liposomal amphotericin b combination (Kar et al., 2021).

Natural products often have relatively poor bioavailability due to a variety of barriers. The lack of bioavailability in these compounds can be due to low absorption, poor solubility, chemical instability, compound size, and poor permeability (Sachdeva et al., 2022). Challenges that have been noted with eugenol specifically are its first pass metabolism and permeability. In a series of papers published by Kar et al. investigators sought to improve the permeability and selectivity (Charan Raja et al., 2021; Kar et al., 2021). To improve these characteristics, the investigators synthesized eugenol oleate, a fatty acid conjugate of eugenol. Their findings support the theory that eugenol oleate increases the selectivity of eugenol. Other eugenol structural conjugates to high biological demand compounds like glycosides, polyamines, or nucleotides may also prove effective in improving selective toxicity.

In the final piece of this investigation, the software Percepta® was used to analyze the eugenol SAR from a pharmacokinetic lens. This *in silico* model predicted that eugenol and the structural analogs tested were pharmacokinetically similar. In comparing the LogP with structures of the eugenol analogs, it was chemically justifiable that 2-methoxyphenol and 2,6-dimethoxyphenol demonstrated lower values due to their increased hydrophilicity. The major weakness of this *in silico* model is that, without experimental data on eugenol or similar compounds, the confidence of the interaction model was very low. All of the predicted CYP interactions (substrate or inhibition) reflected this. Another limitation of this model was that it did not predict the metabolites of the compounds, it only predicted with which enzymes the compounds might interact. A next step towards more accurately predicting the metabolism of eugenol would be to use the same software with experimental data supplementing the predictions. This method would provide higher confidence results providing a more reliable depiction of the metabolism of eugenol. However, the data we received from this model provided a good starting point for future metabolism studies like *in vitro* liver microsome assays.

Based on eugenol’s structure we expect that a major metabolic pathway for this and similar compounds is glucuronidation or sulfonation of the exposed phenolic hydroxyl group. Interestingly, one study found that epoxide formation on the allyl tail followed by hydrolysis was one of the major routes of eugenol metabolism. Notably, this paper estimated that 95% of eugenol was eliminated renally either without modification or though conjugates (Fischer et al., 1990). Deeper investigation into eugenol’s metabolites and their potential toxicity is needed.

In conclusion, eugenol has appreciable antileishmanial activity and some structural modifications to the molecule can improve this activity. Eugenol and its analogs all have similar predicted pharmacokinetic properties but require more precise experimental data to confirm their metabolism. The literature on eugenol and its potential as an antileishmanial agent is incomplete and the data on its analogs is even more sparse. This study provides a springboard for future studies to begin to fill in the gaps in the literature surrounding eugenol as an antileishmanial. More investigation into the mechanism of action, metabolism, and feasibility of eugenol and its analogs is warranted, especially deeper investigation into 4-allylphenol. Not only was 4-allylphenol the most potent of the eugenol analogs tested in this study, its high potency against *L. donovani* axenic amastigotes and selectivity index above 10 warrants further investigation as a lead compound for the treatment of visceral leishmaniasis (Katsuno et al., 2015).

## Data Availability

The original contributions presented in the study are included in the article/Supplementary Material, further inquiries can be directed to the corresponding author.

## Supplementary material

SUPPLEMENTARY FIGURE S1 Dose-response data for eugenol and eugenol structural analogs against L. donovani promastigotes. Dose-response curves were generated and IC50 values calculated using GraphPad Prism version 11. Data from at least 3 experiments are shown.

SUPPLEMENTARY FIGURE S2 Dose-response data for eugenol and eugenol structural analogs against L. donovani amastigotes. Dose-response curves were generated and IC50 values calculated using GraphPad Prism version 11. Data from at least 3 experiments are shown.

SUPPLEMENTARY FIGURE S3 Dose-response data for eugenol and eugenol structural analogs against THP-1 cells. Dose-response curves were generated and LC50 values calculated using GraphPad Prism version 11. Data from at least 3 experiments are shown.

SUPPLEMENTARY TABLE S1 Key reagents used in this investigation with the respective supplier and catalog number included for each compound.

SUPPLEMENTARY TABLE S2 Pharmacokinetic properties of eugenol and its analogs as predicted by percepta.
