# Supplementary material for: Mapping the antileishmanial structure-activity relationship of eugenol
Source: Front Pharmacol. 2026 Sep 30;17:1923146. doi: 10.3389/fphar.2026.1923146 (PMC13661496; doi:10.3389/fphar.2026.1923146)
Supplement: Supplementary file 1 [file Image1.pdf]

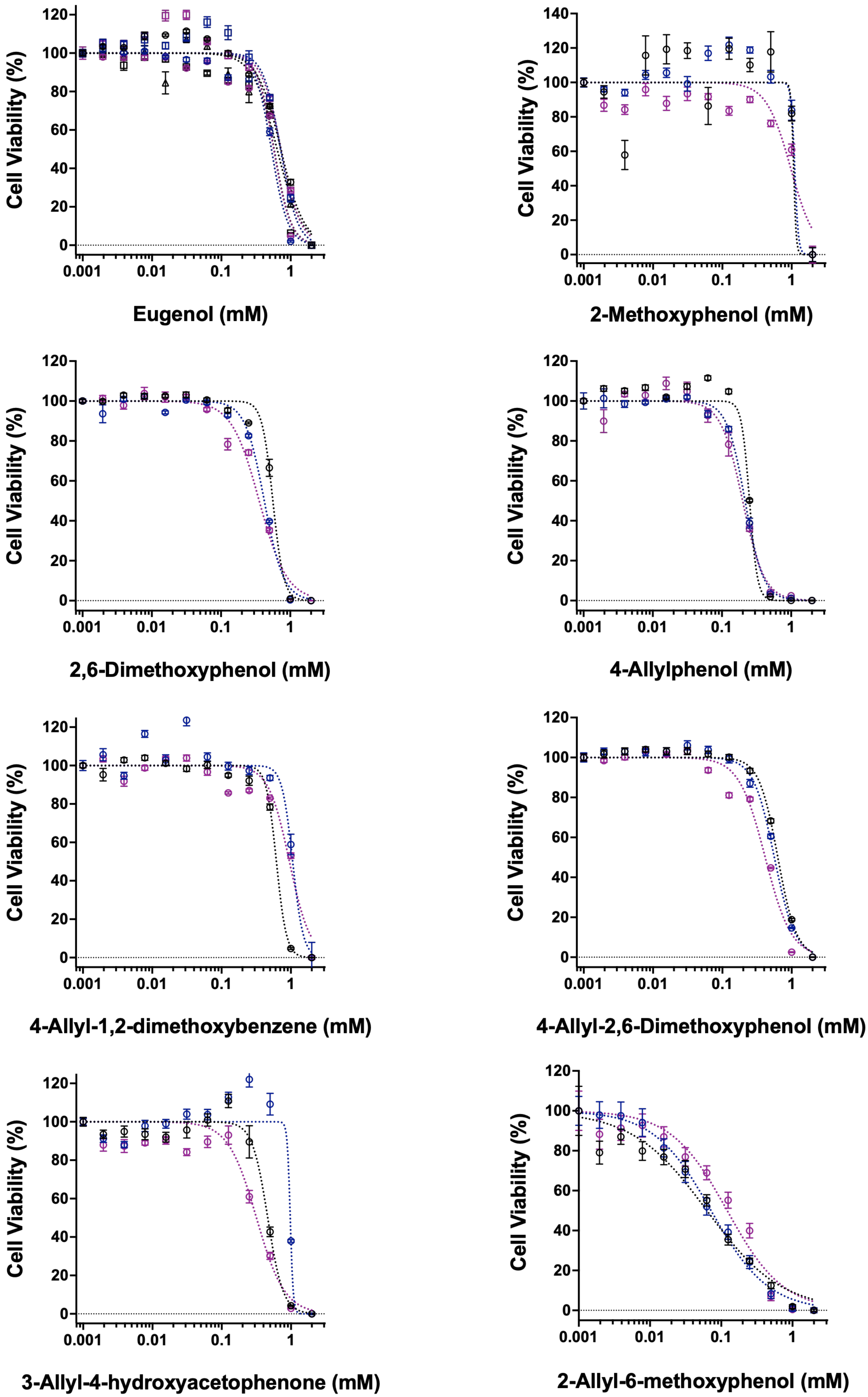
Cell Viability (%)
Eugenol (mM)
Cell Viability (%)
2-Methoxyphenol (mM)
Cell Viability (%)
2,6-Dimethoxyphenol (mM)
Cell Viability (%)
4-Allylphenol (mM)
Cell Viability (%)
4-Allyl-1,2-dimethoxybenzene (mM)
Cell Viability (%)
4-Allyl-2,6-Dimethoxyphenol (mM)
Cell Viability (%)
3-Allyl-4-hydroxyacetophenone (mM)
Cell Viability (%)
2-Allyl-6-methoxyphenol (mM)
0
20
40
60
80
100
120
140
0.001
0.01
0.1
1
