# Supplementary material for: Mapping the antileishmanial structure-activity relationship of eugenol
Source: Front Pharmacol. 2026 Sep 30;17:1923146. doi: 10.3389/fphar.2026.1923146 (PMC13661496; doi:10.3389/fphar.2026.1923146)
Supplement: Supplementary file 2 [file Image2.pdf]

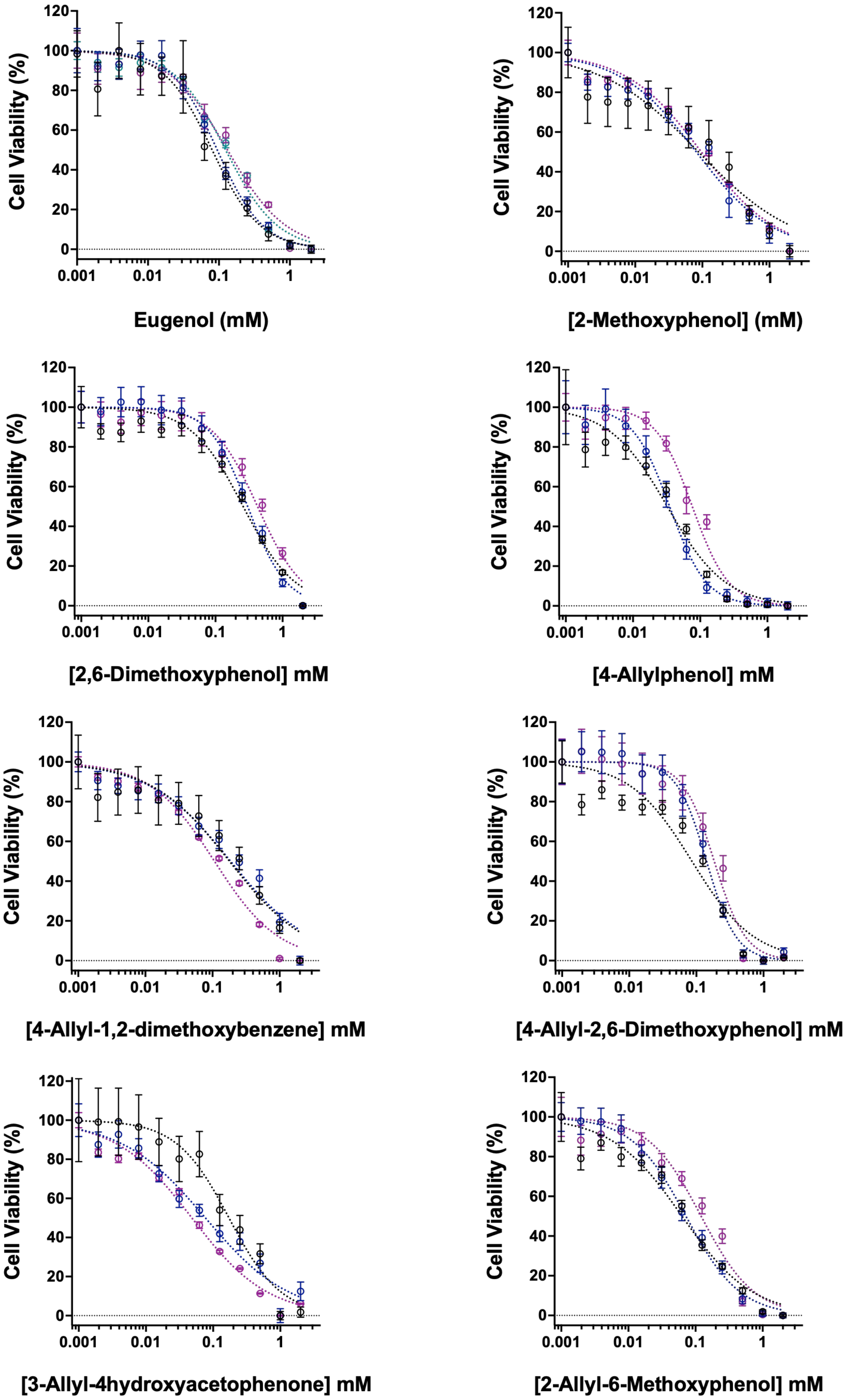
Cell Viability (%)
120
100
80
60
40
20
0
0.001
0.01
0.1
1
Eugenol (mM)
[2-Methoxyphenol] (mM)
[2,6-Dimethoxyphenol] mM
[4-Allylphenol] mM
[4-Allyl-1,2-dimethoxybenzene] mM
[4-Allyl-2,6-Dimethoxyphenol] mM
[3-Allyl-4hydroxyacetophenone] mM
[2-Allyl-6-Methoxyphenol] mM
