# Supplementary material for: Mapping the antileishmanial structure-activity relationship of eugenol
Source: Front Pharmacol. 2026 Sep 30;17:1923146. doi: 10.3389/fphar.2026.1923146 (PMC13661496; doi:10.3389/fphar.2026.1923146)
Supplement: Supplementary file 3 [file Image3.pdf]

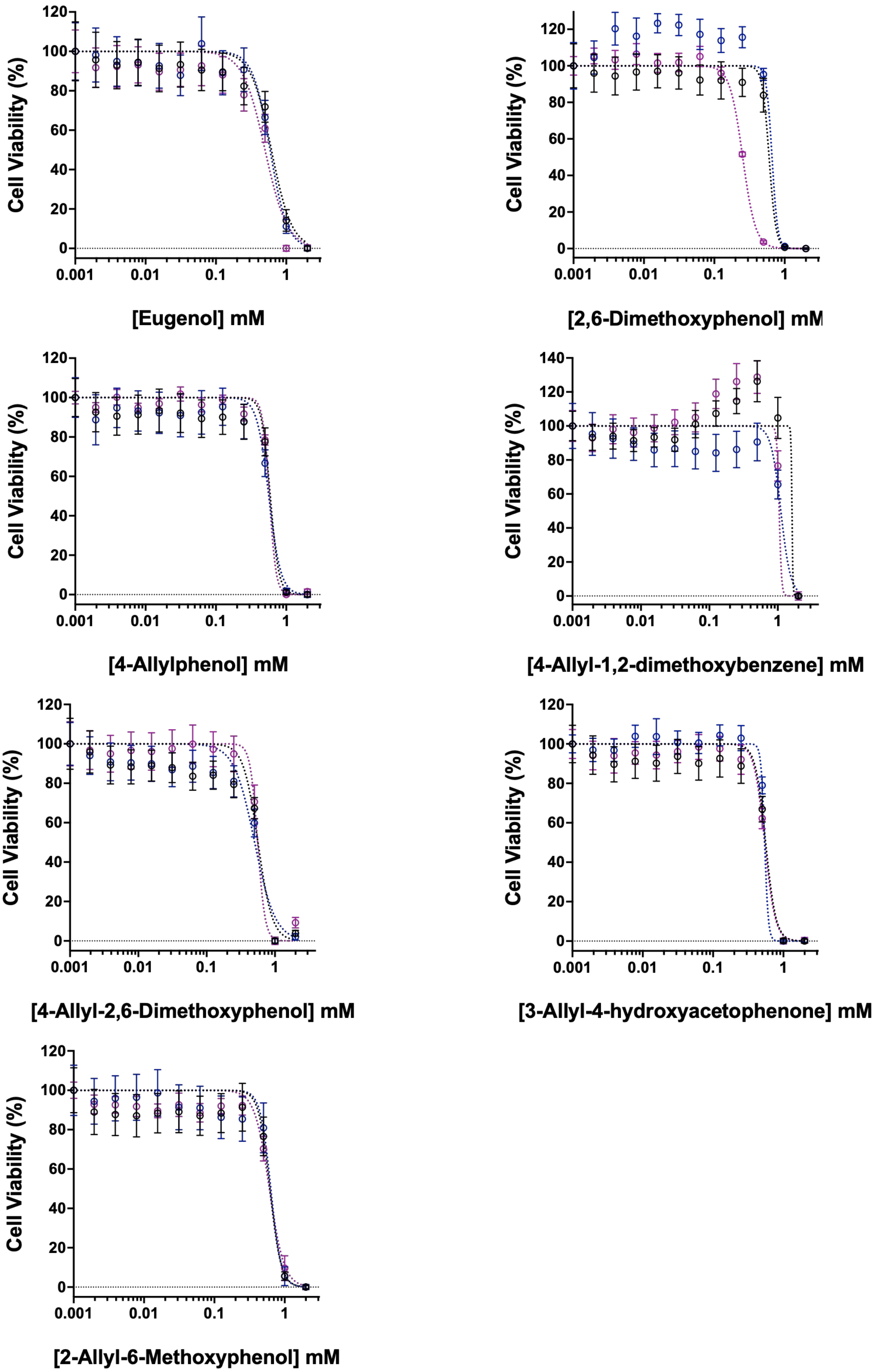
Cell Viability (%)
[Eugenol] mM
[2,6-Dimethoxyphenol] mM
[4-Allylphenol] mM
[4-Allyl-1,2-dimethoxybenzene] mM
[4-Allyl-2,6-Dimethoxyphenol] mM
[3-Allyl-4-hydroxyacetophenone] mM
[2-Allyl-6-Methoxyphenol] mM
