# Supplementary material for: Mapping the antileishmanial structure-activity relationship of eugenol
Source: Front Pharmacol. 2026 Sep 30;17:1923146. doi: 10.3389/fphar.2026.1923146 (PMC13661496; doi:10.3389/fphar.2026.1923146)
Supplement: Supplementary file 4 [file Table1.docx]

Table S1. Key reagents used in this investigation with the respective supplier and catalog number included for each compound

| **Reagent** | **Catalog Number** | **Source** |
| --- | --- | --- |
| *Key Cell Culture and Bioassay Reagents* | | |
| Dulbecco’s Modified Eagle Medium (DMEM) high glucose powder | 12100-061 | Thermo Fisher Scientific, Waltham, MA, USA |
| RPMI 1640 powder | R7755-10L | Sigma Aldrich, St. Louis, MO, USA |
| Fetal Bovine Serum | FBS002 | Neuromics, Edina, MN, USA |
| Penicillin-Streptomycin (10,000 U/mL) | 15140-122 | Thermo Fisher Scientific |
| L-Glutamine (200 mM) | 25030-081 | Thermo Fisher Scientific |
| RPMI Vitamins Solution (100x) | R7256-100mL | Sigma Aldrich |
| RPMI 1640 Amino Acids Solution (50×) | R7131-100mL | Sigma Aldrich |
| Resazurin | TS41890-0050 | VWR International, Radnor, PA, USA |
| *Compounds Evaluated in this Study* |  |  |
| ^A^ Eugenol, PESTANAL^®^, analytical standard | 35995-250mg | Sigma Aldrich |
| ^B^ Eugenol, *ReagentPlus*^®^, 99% | E51791-5G | Sigma Aldrich |
| 2-Allyl-6-methylphenol | A34007-5G | Sigma Aldrich |
| 4-Allyl-1,2-dimethoxybenzene | 284424-5G | Sigma Aldrich |
| 2,6-Dimethoxyphenol | D135550-25G | Sigma Aldrich |
| 4-Allyl-2,6-Dimethoxyphenol | B21981-03-1g | Thermo Fisher Scientific |
| 2-Methoxyphenol | 8183390005 | Sigma Aldrich |
| 4-Allylphenol, Chavicol | AMBH97F05A10-100MG | Sigma Aldrich |
| 3-Allyl-4-hydroxyacetophenone | 632759-5G | Sigma Aldrich |
