# Supplementary material for: Mapping the antileishmanial structure-activity relationship of eugenol
Source: Front Pharmacol. 2026 Sep 30;17:1923146. doi: 10.3389/fphar.2026.1923146 (PMC13661496; doi:10.3389/fphar.2026.1923146)
Supplement: Supplementary file 5 [file Table2.docx]

Table S2. Pharmacokinetic properties of eugenol and its analogs as predicted by Percepta.

| Compound | LogP | Protein Binding % | CYP Inhibition | CYP Substrates |
| --- | --- | --- | --- | --- |
| Eugenol | 2.48 | 75.15 (RI=0.34) | None (low probability) | 1A2 (Low R) |
| 4-Allyl-1,2-Dimethoxybenzene | 2.74 | 81.87 (RI=0.58) | 2C19 (low R) | 1A2 (Low R) |
| 4-Allyl-2,6-Dimethoxyphenol | 2.19 | 61.88 (RI=0.41) | None (low probability) | 1A2 (Low R) |
| 2,6-Dimethoxyphenol | 1.12 | 63.93 (RI=0.29) | None (low probability) | 1A2 (Low R) |
| 2-Methoxyphenol | 1.34 | 74.51 (RI=0.4) | None (low probability) | None |
| 4-Allylphenol | 2.52 | 75.87 (RI=0.57) | None (low probability) | 1A2 (Low R) |
| 2-Allyl-6-Methoxyphenol | 2.48 | 75.14 (RI=0.34) | None (low probability) | 1A2 (Low R) |
| 3-Allyl-4-Hydroxyacetophenone | 2.51 | 78.04 (RI=0.55 | None (low probability) | 1A2 (Low R) |
